# First-in-human phase I/II, open-label study of mRNA-2416 alone or combined with durvalumab in patients with advanced solid tumors and ovarian cancer

**DOI:** 10.1093/oncolo/oyaf115

**Published:** 2025-06-14

**Authors:** Ryan J Sullivan, Oladapo O Yeku, Deanna Teoh, Shilpa Gupta, Daniela Matei, Andressa S Laino, Jing Sun, Lili Zhu, Linh Van, Stephanie Pascarella, Sima J Zacharek, Khanh T Do, Antonio Jimeno

**Affiliations:** Department of Medicine, Massachusetts General Hospital, Boston, MA, USA; Department of Medicine, Massachusetts General Hospital, Boston, MA, USA; Department of Obstetrics, Gynecology and Women’s Health, University of Minnesota Masonic Cancer Center, Minneapolis, MN, USA; Hematology and Medical Oncology, Cleveland Clinic Taussig Cancer Institute, Cleveland, OH, USA; Department of Obstetrics and Gynecology, Northwestern University, Chicago, IL, USA; Oncology Research and Clinical Development, Oncology, Moderna Therapeutics, Inc., Cambridge, MA, USA; Oncology Research and Clinical Development, Oncology, Moderna Therapeutics, Inc., Cambridge, MA, USA; Oncology Research and Clinical Development, Oncology, Moderna Therapeutics, Inc., Cambridge, MA, USA; Oncology Research and Clinical Development, Oncology, Moderna Therapeutics, Inc., Cambridge, MA, USA; Oncology Research and Clinical Development, Oncology, Moderna Therapeutics, Inc., Cambridge, MA, USA; Oncology Research and Clinical Development, Oncology, Moderna Therapeutics, Inc., Cambridge, MA, USA; Oncology Research and Clinical Development, Oncology, Moderna Therapeutics, Inc., Cambridge, MA, USA; Medical Oncology, University of Colorado Cancer Center, Aurora, CO, USA

**Keywords:** advanced solid tumors, ovarian cancer, combination immunotherapy, mRNA therapy, checkpoint blockade, PD-1

## Abstract

**Background:**

mRNA-2416 is a novel lipid nanoparticle-encapsulated messenger RNA (mRNA) encoding human OX40 ligand (OX40L) for intratumoral (Itu) injection. OX40L plus immune checkpoint inhibitor (ICI) increased preclinical antitumor activity, thus mRNA-2416 plus ICI may potentiate antitumor activity.

**Methods:**

This first-in-human, phase I/II, open-label, multicenter study examined the safety, tolerability, and efficacy of mRNA-2416 alone (arm A) or with durvalumab (arm B) in patients with advanced solid tumors or lymphoma (NCT03323398). Phase I primary objectives included assessment of safety/tolerability and maximum tolerated dose (MTD)/recommended dose for expansion; phase II arm B dose expansion assessed objective response rate in ovarian cancers. Secondary objectives included pharmacokinetics, disease control rate, duration of response, and progression-free survival (PFS). Assessments of immunologic response to treatment were exploratory.

**Results:**

From August 2017 to August 2021, 79 patients were enrolled; 61 received treatment (arm A: 39, arm B: 22), including 16 in the expansion cohort. MTD was not reached. Treatment-related emergent adverse events were primarily grade 1/2, with 8 grade 3 and no grade 4/5 events. On-treatment tumor biopsies demonstrated increased OX40L protein expression, elevated PD-L1, and proinflammatory responses. Tumor shrinkage occurred in injected and surrounding non-injected tumors. Median (95% CI) PFS was 60.0 (50.0 to 108.0) and 50.0 (38.0 to 55.0) days for arms A and B, respectively.

**Conclusions:**

mRNA-2416 alone or with durvalumab was well tolerated. Pharmacodynamic analyses support Itu mRNA proof-of-concept. Predefined primary efficacy endpoints were not met in an exploratory cohort of ovarian cancer. Additional research is warranted to further inform this therapeutic approach.

**Implications for Practice**

This study aimed to demonstrate that expression of mRNA-encoded human OX40L (mRNA-2416) via intratumoral injection could be a potential route to enhance antitumor immunity. mRNA-2416 was tolerable with an observed safety profile similar to other OX40 pathway therapeutics and was associated with an increase in OX40L protein expression in injected tumors. Patients treated with mRNA-2416 showed systemic cytokine responses, upregulation of antitumor immunity markers and PD-L1 expression in tumor biopsies, and evidence of tumor shrinkage. This study provided proof of concept for the mechanism of action of the first-in-class mRNA-2416 based on tumor biopsies, warranting further research into mRNA-based therapeutics in advanced solid tumors and ovarian cancer.

## Background

Immune checkpoint inhibitors (ICIs) have substantially improved clinical outcomes in several malignancies.^1,2^ However, many tumors do not respond to treatment, or become resistant to, ICIs.^3,4^ In particular, recurrent/refractory ovarian cancer has shown limited response to ICI monotherapy, with response rates of <15%.^5^ Thus, there is an urgent need to identify anticancer agents capable of augmenting the effects of ICIs to improve response.

**Figure 1.**
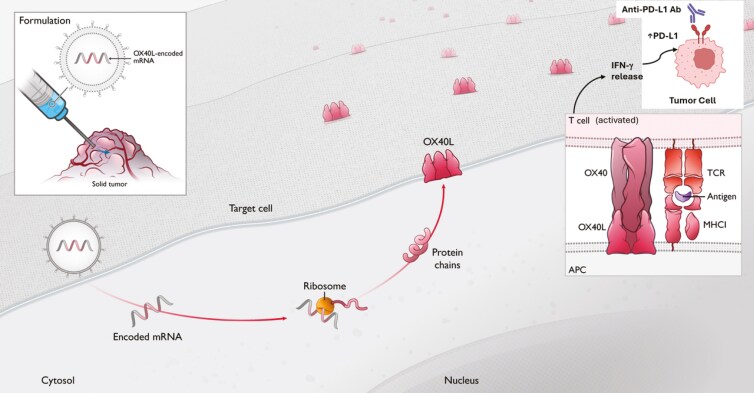
*Mechanism of action of mRNA-2416*. Direct injection of mRNA-2416 into a tumor results in cellular expression of OX40L protein, a transmembrane co-stimulatory molecule that is normally expressed on antigen-presenting cells (APCs). OX40 is induced on activated T cells upon antigen stimulation, while OX40L expression on mature APCs prevents T-cell anergy and promotes effector expansion and memory differentiation. OX40L when bound to its receptor (OX40) can promote effector T-cell proliferation in the presence of a recognized antigen [inset bottom right]. Heightened signaling promotes the release of cytokines, particularly interferon-gamma (IFN-γ), from lymphocytes such as T cells and NK cells within the TME. In turn, IFN-γ stimulates the upregulation of PD-L1, an IFN-inducible gene, on both tumor cells [inset top right] and tumor-associated immune cells.

The OX40 receptor (tumor necrosis factor receptor superfamily 4, CD134) is expressed on activated immune effector cells such as cytotoxic and helper T cells and natural killer cells.^6^ Binding of the OX40 receptor with the OX40 ligand (OX40L) in the presence of a recognized tumor neoantigen enhances the expansion of CD4 + and CD8 + T cells and increases T-cell memory responses while inhibiting T regulatory cells.^7^ Analyses of tissue microarrays in tumor samples of NSCLC and advanced ovarian malignancies revealed that although OX40 is abundantly present in immune cells within the tumor microenvironment, OX40L expression is limited.^8^ This imbalance likely restricts effective OX40 pathway engagement, thereby impairing optimal T-cell activation and antitumor responses. mRNA-2416 is a novel lipid nanoparticle-encapsulated mRNA encoding human OX40L, which was developed for intratumoral injection (Figure 1).^9^ Induction of OX40L within the tumor microenvironment through direct injection of mRNA-2416 has the potential to restore OX40 receptor engagement and enhance antitumor immunity through stimulation of tumor-specific T-cell activation, resulting in local and systemic antitumor responses. In murine models, durable tumor regressions have been observed with a single-agent OX40 agonist in combination with programmed cell death protein 1 (PD-1) blockade^10,11^ and antitumor activity was shown with approaches using mRNA encoding OX40L.^12^ Thus, induction of OX40L expression is a potential strategy for enhancing antitumor immunity through stimulation of tumor-specific T-cell activation, resulting in local and systemic antitumor responses.

We report data from the first-in-human phase I/II study of mRNA-2416, alone and in combination with durvalumab, in patients with advanced solid tumors and in patients with platinum-resistant ovarian cancers in the follow-up phase II study. The primary objectives were safety and tolerability in the phase I study and antitumor activity in the phase II study.

## Methods

### Study design

This was an open-label, multicenter, phase I/II study designed to evaluate repeated intratumoral injections of mRNA-2416 alone (arm A) and in combination with intravenous durvalumab (arm B) in patients with advanced solid tumors or lymphoma (NCT03323398; retrospectively registered October 25, 2017). The study was conducted from August 2017 to August 2021 and included dose escalation, dose confirmation, and dos-expansion parts (Supplementary Figure S1).

#### Dose escalation and confirmation

The phase I, 3 + 3 dose escalation and confirmation study was designed to assess safety and tolerability and define the maximum tolerated dose (MTD) and recommended dose for expansion (RDE), as defined in Supplementary Table S1 for mRNA-2416 alone (arm A) and mRNA-2416 combined with durvalumab (arm B). For arm A, the starting mRNA-2416 dose was 1.0 mg, with subsequent escalation doses of 2.0, 4.0, and 8.0 mg. Once the expected MTD/RDE was cleared in arm A, dose escalation started for arm B at 4.0 mg mRNA-2416 combined with 1500 mg durvalumab, with a subsequent escalation dose of 8.0 mg mRNA-2416 combined with 1500 mg durvalumab. Further details regarding dose escalation and confirmation are provided in the Supplementary Text.

#### Dose expansion

Dose expansion was not pursued in arm A due to the modest efficacy of mRNA-2416 monotherapy; therefore, dose expansion was only conducted in arm B because of the hypothesized biological synergy in combination with ICIs, based on preclinical studies. The phase I dose expansion study was designed to assess the objective response rate (ORR) of mRNA-2416 combined with durvalumab in patients with ovarian cancer at the MTD/RDE determined in the dose escalation study. Additional details regarding the dose-expansion phase are provided in the Supplementary Text.

#### mRNA-2416 and durvalumab administration

All patients were required to have a tumor(s) of sufficient size to support injection of the first planned mRNA-2416 dose directly into the tumor. If the tumor regressed, and the size of the accessible tumor would not support the planned dose, then the next lowest dose supported by the tumor size was administered following documented approval from the sponsor. Durvalumab was administered as an intravenous infusion at a fixed dose of 1500 mg every 4 weeks for patients weighing ≥30 kg and weight-based dosing of 20 mg/kg for patients weighing <30 kg. The standard infusion time was 1 hour. For patients receiving mRNA-2416 in combination with durvalumab, the intratumoral injection of mRNA-2416 was administered first followed by durvalumab infusion, which was administered ≥30 minutes later.

#### Endpoints

The primary endpoints of the phase I part of the study were to determine the safety and tolerability of mRNA-2416 alone and when combined with durvalumab via the incidence and nature of dose-limiting toxicities (DLTs) and the incidence, nature, and severity of adverse events (AEs). In the phase II part of the study, the primary endpoint was the ORR with mRNA-2416 alone and in combination with durvalumab. The secondary endpoint for the phase I part of the study was ORR, and for the phase II part of the study was disease control rate; other secondary endpoints for both studies included progression-free survival (PFS), duration of response, presence and/or concentration of antibodies against OX40L, and pharmacokinetics. Exploratory endpoints for both studies included assessments of immunologic response to treatment.

### Patients

Eligible patients were aged ≥18 years; had histologically or cytologically confirmed advanced/metastatic solid tumors or lymphoma that progressed on or were intolerant to, all approved therapies; had ≥1 measurable lesion as defined by Response Evaluation Criteria in Solid Tumors (RECIST) v1.1 and one accessible non-visceral lesion (dose escalation part) or one visceral lesion (dose confirmation part) for intratumoral injection; had an Eastern Cooperative Oncology Group performance status of ≤1; had adequate hematological, hepatic, renal, and thyroid function; had a life expectancy of ≥12 weeks; and weighed ≥30 kg. Specific for the dose expansion, patients had confirmed diagnosis of ovarian cancer and had received ≥2 prior lines of therapy. Additionally, patients with a known breast cancer (*BRCA*) mutation were required to have been treated with and progressed on prior poly (ADP-ribose) polymerase inhibitor, have ≥1 lesion amenable to injection, and have a biopsy from a site that had not been previously irradiated. There was no limit on the number of prior regimens and use of prior ICIs was not excluded.

### Assessments

#### Safety and clinical response

The incidence and nature of DLTs (Supplementary Table S1), AEs, and serious AEs according to the National Cancer Institute (NCI) Common Terminology Criteria for Adverse Events version 4.03 were recorded. ORR based on RECIST v1.1 was defined as the proportion of patients with partial response (PR) or better. PR or complete response could be confirmed or unconfirmed. Disease control rate was defined as the proportion of patients with the best overall response of PR or better, or stable disease (SD) for ≥55 days from the date of first dose. PFS was defined as the time from the first dose to the first occurrence of disease progression or death, whichever occurred sooner. The disease control rate was based on RECIST v1.1, while ORR and PFS were based on RECIST v1.1 and immune-related response criteria (irRC) or Lymphoma Response to Immunomodulatory Therapy Criteria (LYRIC).

#### Biomarkers

Immune responses were studied in the peripheral circulation and in tumor biopsy specimens. Formalin-fixed, paraffin-embedded (FFPE) tumor biopsy specimens were examined using immunohistochemistry (IHC), multiplexed quantitative immunofluorescence (mQIF), and/or bulk RNA sequencing. PD-L1 IHC was carried out using the SP263 clone and scored semi-quantitatively for expression in tumor and immune cell populations. mQIF analyses were performed using 2 monoclonal antibodies: D6K7R (OX40L; Cell Signaling Technologies) and LN10 (CD3; Leica); tumor versus non-tumor expression was assessed using AE1/AE3 (pan-cytokeratin to demarcate epithelial populations; Agilent). Tumor slides were stained with antibodies and fluorescent dyes on the Leica Bond RX; stained slides were then scanned on the Vectra Polaris and images were quantified using Automated Quantitative Analysis (AQUA^©^). Cytokines were evaluated in both tumor tissues and plasma using RNA sequencing and/or electrochemiluminescence assays (V-PLEX Proinflammatory Panel 1 Human Kit, MSD). For protein analyses, fold change was calculated relative to baseline values. For the ovarian cancer patient who achieved PR, the screening sample was missing for cytokine analysis, and the median of all baseline values was used for fold change calculation; for values below the limit of quantification, the reference value for limit of detection divided by 2 was used for fold change analysis. For RNAseq analyses, excess non-tumor tissue (eg, skeletal muscle, fat, skin) was removed if present in FFPE blocks, and total RNA extracted from FFPE curls totaled approximately 0.2 mm^3^ of enriched tumor and surrounding tumor-associated stroma. Libraries were generated using a stranded RNAseq kit (KAPA) using PCR Allele Competitive Extension capture probes (Personalis), and sequencing was carried out using a HiSeq instrument (Illumina) at a sequencing output of ≥100 M total reads (125 bp paired-end reads) per sample. RNAseq analytics was processed through the Personalis ACE Transcriptome platform and gene expression was quantified with raw count and transcripts per kilobase million (TPM). The gene expression profile (GEP) score of the T-cell inflamed signature was calculated as a weighted sum of normalized expression values for the 18 signature genes.^13,14^ For housekeeping normalization, raw counts for the individual genes were log_10_ transformed and then normalized by subtracting the arithmetic mean of the log_10_ counts for a set of 11 housekeeping genes. Cytolytic activity was calculated as the geometric mean of GZMA and PRF1 expression with TPM.^15^

### Pharmacokinetics

Serum, plasma, and whole blood samples were collected at designated timepoints during treatment cycles to assess serum mRNA concentrations. The individual serum, plasma, and whole blood concentrations versus actual time data for mRNA-2416 were used to derive pharmacokinetic parameters using non-compartmental analyses with Phoenix WinNonlin version 8.4 or higher. Pharmacokinetic parameters included maximum observed concentration (C_max_), time to peak concentration (t_max_), the area under the curve (AUC) over a dosing interval (AUC_0−t_), AUC for total drug exposure across time (AUC_0−inf_), %AUC, and elimination half-life (t_1/2_).

### Statistical analysis

All analyses were conducted using SAS software (SAS Institute, Inc.) version 9.4 or higher. No formal sample size estimation was performed, and the number of patients was chosen based on a 3 + 3 dose escalation design. Safety analyses were conducted in the population of enrolled patients who received any amount of study drug (safety population). All enrolled patients who received any amount of the study drug and had ≥1 tumor response evaluation were included in efficacy analyses based on the dose of mRNA-2416 received. ORR and disease control rate were summarized with Clopper–Pearson 95% confidence intervals (CIs). PFS was summarized using descriptive statistics (mean, 95% CI, minimum, and maximum), and the Kaplan–Meier method was used to estimate the distribution functions of PFS per RECIST v1.1 and irRC.

## Results

### Patient disposition and characteristics

**Figure 2.**
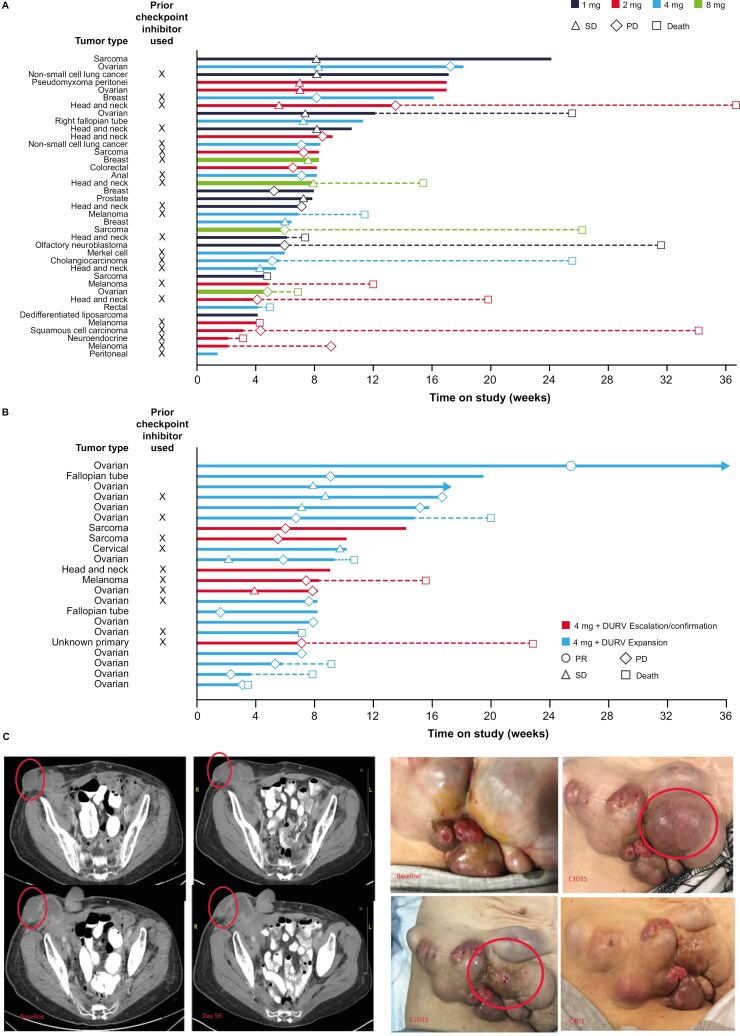
Patient level trajectories over time based on RECIST v1.1. Swimmer plots for patients in (A) arm A and (B) arm B. (C) Evidence of tumor shrinkage in patients with serous fallopian/ovarian cancer. DURV, durvalumab; PD, progressive disease; PR, partial response; SD, stable disease.

From August 2017 to August 2021, a total of 79 patients were enrolled in the study and 61 received treatment (arm A: 39; arm B: 22), including 16 patients in the expansion cohort. Of the 61 patients who received mRNA-2416, 1 completed mRNA-2416 treatment, and of the 22 patients who received mRNA-2416 + durvalumab, 2 (9%) completed durvalumab treatment (Supplementary Figures S2A and S2B). Figure 2A and B show the time on study for patients in each arm. The mean (± standard deviation) mRNA-2416 treatment durations for arms A and B were 5.8 (±4.9) weeks and 7.1 (±4.6) weeks, respectively. Demographics and clinical characteristics of the patients enrolled in the phase I study are shown in Table 1. Median (range) age was 65 (23-82) years and the most frequent tumor types included ovarian (30%), head and neck squamous cell carcinoma (15%), and sarcoma (10%); no patients had lymphoma. Overall, nearly half of patients had received prior ICIs (49% in arm A; 41% in arm B) and most patients had received at least one prior cancer therapy (94.9% in arm A; 100% in arm B).

**Table 1.** Demographic and clinical characteristics for patients by arm and overall.

Characteristic	Phase I study		Overall population^*^ (*N* = 61)
	Arm A (*n* = 39)	Arm B (*n* = 22)	
Age, years, median (range)	66 (23-82)	63 (28-78)	65 (23-82)
Female sex, *n* (%)	18 (46)	18 (82)	36 (59)
Race, *n* (%)			
Black	—	1 (5)	1 (2)
Other	4 (10)	2 (9)	6 (10)
White	35 (90)	19 (86)	54 (89)
Ethnicity, *n* (%)			
Hispanic or Latino	4 (10)	0 (0)	4 (7)
Not Hispanic or Latino	35 (90)	22 (100)	57 (93)
Time since initial diagnosis to first dose, months, median (range)^†^	45.2 (6.1-225.1)	64.9 (13.6-213.2)	58.0 (6.1-225.1)
Cancer types, *n* (%)^‡^			
Anal	1 (3)	0 (0)	1 (2)
Breast	4 (10)	0 (0)	4 (7)
Cervical	0 (0)	1 (5)	1 (2)
Colorectal	1 (3)	0 (0)	1 (2)
HNSCC	8 (21)	1 (5)	9 (15)
Melanoma	4 (10)	1 (5)	5 (8)
NSCLC	2 (5)	0 (0)	2 (3)
Ovarian	4 (10)	14 (64)	18 (30)
Prostate	1 (3)	0 (0)	1 (2)
Rectal	1 (3)	0 (0)	1 (2)
Sarcoma	4 (10)	2 (9)	6 (10)
Other^§^	9 (23)	3 (14)	12 (20)
ECOG performance status, *n* (%)			
0	10 (26)	5 (23)	15 (25)
1	27 (69)	17 (77)	44 (72)
2	2 (5)	—	2 (3)

^*^Overall population represents patients from both arms and studies.

^†^Time from initial diagnosis to first dose in months is calculated as (date of first dose of study drug − date of initial diagnosis + 1)/30.4375.

^‡^Only patients with fallopian/ovarian cancer were enrolled in the expansion study.

^§^Other cancer types included squamous cell carcinoma unknown primary (*n* = 2), fallopian tube carcinoma (*n* = 3), olfactory neuroblastoma (*n* = 1), dedifferentiated liposarcoma (*n* = 1), pseudomyxoma peritonei (*n* = 1), neuroendocrine carcinoma (*n* = 1), cholangiocarcinoma (*n* = 1), peritoneal adenocarcinoma (*n* = 1), and Merkel cell carcinoma (*n* = 1).

ECOG, Eastern Cooperative Oncology Group; HNSCC, head and neck squamous cell carcinoma; NSCLC, non-small cell lung cancer.

Demographics and clinical characteristics of the patients with ovarian cancer enrolled in the phase II expansion study are shown in Supplementary Table S2.

### Safety

Safety results are presented as the cumulative number of AEs over phases I and II (Table 2 and Supplementary Tables S3-S6). There were no DLTs at any tested dose level and the MTD was not reached for either arm. Most AEs attributed to mRNA-2416 were grade 1/2. In arm A, the most frequent mRNA-2416-related treatment-emergent AEs (TEAEs) occurring in ≥10% of patients included injection site pain (28%), flushing (26%), pyrexia (23%), fatigue (21%), injection site erythema (21%), nausea (18%), chills (15%), myalgia (13%), dyspnea (13%), back pain (10%), and influenza-like illness (10%; Table 2). In arm B, the most frequent mRNA-2416-related TEAEs occurring in ≥10% of patients included pyrexia (46%), injection site pain (41%), nausea (32%), fatigue (27%), flushing (23%), chills (14%), influenza-like illness (14%), tachycardia (14%), hypotension (14%), dyspnea (14%), and hyperhidrosis (14%; Table 2); the most frequent durvalumab-related TEAEs occurring in ≥10% of patients were fatigue (27%), pyrexia (23%), and nausea (23%; Supplementary Table S4). Of the patients who experienced ≥1 serious TEAE, 5 were considered to be related to mRNA-2416: grade 3 skin ulceration, grade 3 dyspnea, grade 2 injection-related reaction with hypoxia, and grade 2 systemic inflammatory response syndrome in arm A; and a grade 3 cerebrovascular accident with positive lupus anticoagulant assessed by the investigator as being possibly related to both mRNA-2416 and durvalumab treatment in arm B. Additional details regarding AEs by treatment arm are described in Table 2 and Supplementary Tables S3-S6.

**Table 2.** Treatment-related TEAEs by preferred term occurring with frequency ≥5% for arm A or B.

	Arm A							Arm B					
	Dose escalation					Dose confirmation	Total (*N* = 39)	Dose escalation	Dose confirmation	Dose expansion			Total (*N* = 22)
	1 mg (*n* = 11)	2 mg (*n* = 12)	4 mg (*n* = 12)	8 mg (*n* = 3)	Sub-total (*n* = 38)	8 mg (*n* = 1)		4 mg (*n* = 3)	4 mg (*n* = 3)	2 mg (*n* = 1)	4 mg (*n* = 15)	Sub-total (*n* = 16)	
*Patients with ≥1 mRNA-2416-related TEAE*	6 (54.5)	11 (91.7)	11 (91.7)	3 (100)	31 (81.6)	0	31 (79.5)	1 (33.3)	3 (100)	1 (100)	12 (80.0)	13 (81.3)	17 (77.3)
Injection site pain	1 (9.1)	5 (41.7)	5 (41.7)	0	11 (28.9)	0	11 (28.2)	1 (33.3)	0	1 (100)	7 (46.7)	8 (50.0)	9 (40.9)
Flushing	2 (18.2)	3 (25.0)	4 (33.3)	1 (33.3)	10 (26.3)	0	10 (25.6)	0	2 (66.7)	0	3 (20.0)	3 (18.8)	5 (22.7)
Pyrexia	2 (18.2)	3 (25.0)	3 (25.0)	1 (33.3)	9 (23.7)	0	9 (23.1)	0	2 (66.7)	0	8 (53.3)	8 (50.0)	10 (45.5)
Fatigue	1 (9.1)	2 (16.7)	4 (33.3)	1 (33.3)	8 (21.1)	0	8 (20.5)	0	1 (33.3)	0	5 (33.3)	5 (31.3)	6 (27.3)
Injection site erythema	0	4 (33.3)	2 (16.7)	2 (66.7)	8 (21.1)	0	8 (20.5)	1 (33.3)	0	0	1 (6.7)	1 (6.3)	2 (9.1)
Nausea	2 (18.2)	1 (8.3)	4 (33.3)	0	7 (18.4)	0	7 (17.9)	1 (33.3)	1 (33.3)	0	5 (33.3)	5 (31.3)	7 (31.8)
Chills	1 (9.1)	2 (16.7)	3 (25.0)	0	6 (15.8)	0	6 (15.4)	0	1 (33.3)	0	2 (13.3)	2 (12.5)	3 (13.6)
Myalgia	0	1 (8.3)	4 (33.3)	0	5 (13.2)	0	5 (12.8)	0	0	0	1 (6.7)	1 (6.3)	1 (4.5)
Dyspnea	0	2 (16.7)	3 (25.0)	0	5 (13.2)	0	5 (12.8)	0	1 (33.3)	0	2 (13.3)	2 (12.5)	3 (13.6)
Back pain	0	1 (8.3)	3 (25.0)	0	4 (10.5)	0	4 (10.3)	0	0	0	0	0	0
Influenza-like illness	0	1 (8.3)	2 (16.7)	1 (33.3)	4 (10.5)	0	4 (10.3)	0	0	0	3 (20.0)	3 (18.8)	3 (13.6)
Vomiting	0	0	3 (25.0)	0	3 (7.9)	0	3 (7.7)	0	0	0	2 (13.3)	2 (12.5)	2 (9.1)
Abdominal pain	1 (9.1)	0	2 (16.7)	0	3 (7.9)	0	3 (7.7)	0	0	0	1 (6.7)	1 (6.3)	1 (4.5)
Decreased appetite	0	1 (8.3)	1 (8.3)	1 (33.3)	3 (7.9)	0	3 (7.7)	0	0	0	2 (13.3)	2 (12.5)	2 (9.1)
Injection site pruritus	1 (9.1)	0	2 (16.7)	0	3 (7.9)	0	3 (7.7)	0	0	0	1 (6.7)	1 (6.3)	1 (4.5)
Injection site discomfort	0	2 (16.7)	0	0	2 (5.3)	0	2 (5.1)	0	1 (33.3)	0	0	0	1 (4.5)
Localized edema	1 (9.1)	1 (8.3)	0	0	2 (5.3)	0	2 (5.1)	0	0	0	0	0	0
Tachycardia	0	1 (8.3)	1 (8.3)	0	2 (5.3)	0	2 (5.1)	0	2 (66.7)	0	1 (6.7)	1 (6.3)	3 (13.6)
Hypoxia	1 (9.1)	0	1 (8.3)	0	2 (5.3)	0	2 (5.1)	0	0	0	0	0	0
Rash erythematous	0	2 (16.7)	0	0	2 (5.3)	0	2 (5.1)	0	0	0	0	0	0
Pruritus	2 (18.2)	0	0	0	2 (5.3)	0	2 (5.1)	0	0	0	2 (13.3)	2 (12.5)	2 (9.1)
Pelvic pain	0	1 (8.3)	1 (8.3)	0	2 (5.3)	0	2 (5.1)	0	0	0	0	0	0
Hypotension	0	0	0	1 (33.3)	1 (2.6)	0	1 (2.6)	0	2 (66.7)	0	1 (6.7)	1 (6.3)	3 (13.6)
Hyperhidrosis	0	1 (8.3)	0	0	1 (2.6)	0	1 (2.6)	0	1 (33.3)	0	2 (13.3)	2 (12.5)	3 (13.6)
Injection site rash	0	1 (8.3)	0	0	1 (2.6)	0	1 (2.6)	0	0	0	2 (13.3)	2 (12.5)	2 (9.1)
Headache	0	0	1 (8.3)	0	1 (2.6)	0	1 (2.6)	0	0	0	2 (13.3)	2 (12.5)	2 (9.1)
Increased aspartate aminotransferase	0	0	0	1 (33.3)	1 (2.6)	0	1 (2.6)	0	0	0	2 (13.3)	2 (12.5)	2 (9.1)

Data are *n* (%). A TEAE is defined as any adverse event that has newly appeared, increased in frequency, or worsened in severity occurring on or after the first dose of the study drug. Patients may have more than one event per system organ class and preferred term. At each level of patient summarization, a patient is counted once if the patient reported one or more events. Adverse events were coded with Medical Dictionary for Regulatory Activities Version 24.1.

TEAE, treatment-emergent adverse event.

### Clinical response

**Figure 3.**
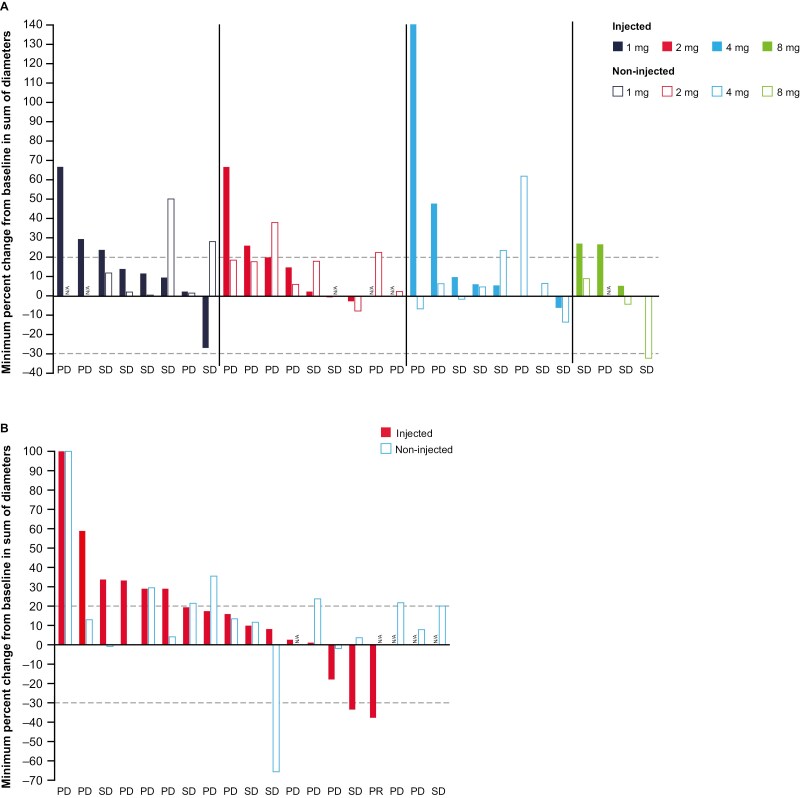
Tumor volume change, using RECIST v1.1, by tumor injection status for patients in (A) arm A (1, 2, 4, or 8 mg mRNA-2416) and (B) arm B (4 mg mRNA-2416 [2 mg mRNA-2416 in 1 patient] + durvalumab). N/A, not available; PD, progressive disease; SD, stable disease.

Tumor shrinkage was observed in several patients from both arms (Figure 3A and B). Based on RECIST v1.1 criteria, ORRs (95% CI) for arms A and B were 0.0% (0.0 to 11.9%) and 5.3% (0.1 to 26.0%), respectively. In arm A (*n* = 29) SD was observed in 9 (31%) patients, progressive disease in 15 (52%) patients, and 5 (17%) patients were not evaluable. In arm B (*n* = 19), 1 patient (5%; 4.0 mg) with ovarian cancer achieved PR but the response was unconfirmed due to the absence of a confirmatory scan; SD was observed in 2 (11%) patients, and progressive disease in 16 (84%) patients. The median PFS for arms A and B were 60 days (95% CI: 50 to 108 days; range: 29 to 179 days) and 50 days (95% CI: 38 to 55 days; range: 11-117 days), respectively (Supplementary Table S7).

Tumor response based on irRC, for patients in arm A, was reported as SD (7 [24%] patients) and progressive disease (16 [55%] patients); 6 (21%) patients were not evaluable. For patients in arm B, PR was observed for 1 (5%) patient with ovarian cancer, SD for 2 (11%), progressive disease for 15 (79%), and 1 (5%) patient was not evaluable (Supplementary Table S7).

One notable tumor response was seen in a 63-year-old patient with serous fallopian/ovarian cancer who had received 13 prior lines of systemic therapy (Figure 2C). While this patient had the overall best response of SD by RECIST, the injected lesion (indicated by the circle in Figure 2C) regressed to near resolution after four injections; further, surrounding non-injected lesions visibly flattened by cycle 4.

### Biomarker assessments

**Figure 4.**
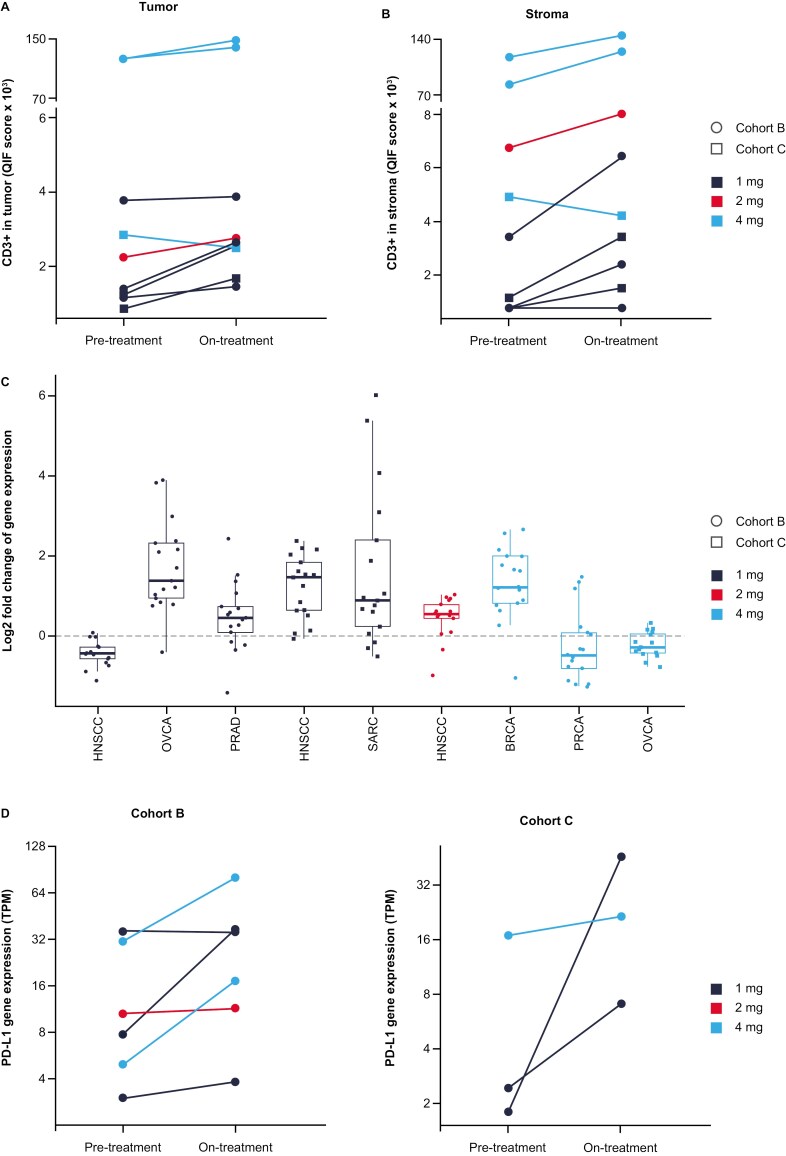
Immunomodulatory effects of mRNA-2416 monotherapy treatment. Protein-level changes in CD3 + following mRNA-2416 monotherapy were calculated in (A) tumor and (B) stroma tissues from patients in the B (on-treatment biopsy 24-48 hours post-C1D1) and C (on-treatment biopsy 24-48 hours post-C2D1) biopsy groups (site of injection; *n* = 9). (C) Gene expression profile of T-cell-inflamed signature genes. Log_2_ fold change (LFC) of mRNA expression as assessed by RNAseq. Each dot represents the LFC of an individual positive association gene in the gene expression profile score (*n* = 17 genes; 1 gene was not evaluable). (D) mRNA expression levels of PD-L1 pretreatment and on-treatment. BRCA, breast cancer; C1D1, cycle 1, day 1; OVCA, ovarian cancer; QIF, quantitative immunofluorescence; PRAD, prostate adenocarcinoma; PRCA, prostate cancer; SARC, sarcoma; TPM, transcripts per kilobase million.

Patients treated with mRNA-2416 as a monotherapy displayed increased OX40L protein in the tumor microenvironment (TME) after injection (Supplementary Figures S3A and S3B). Additionally, confirmed increases in OX40L protein expression relative to baseline were seen in 4/4 biopsies evaluated from mRNA-2416-injected tumors 24-48 hours post-treatment in patients from arm B (Supplementary Figure S3C). Pharmacodynamic effects of mRNA-2416 treatment included increased T-cell presence in the TME in both tumor nests and surrounding stroma as assessed by mQIF (Figure 4A and B), and activation of a proinflammatory gene expression response (assessed by GEP score using previously described methods),^14^ including overall upregulation of T-cell-inflamed signature genes in 6/9 monotherapy patients assessed (Figure 4C). PD-L1, an IFN-inducible gene, was also elevated after treatment (Figure 4D), as measured by RNAseq. These effects were most pronounced in injected tumors (Supplementary Figure S4).

**Figure 5.**
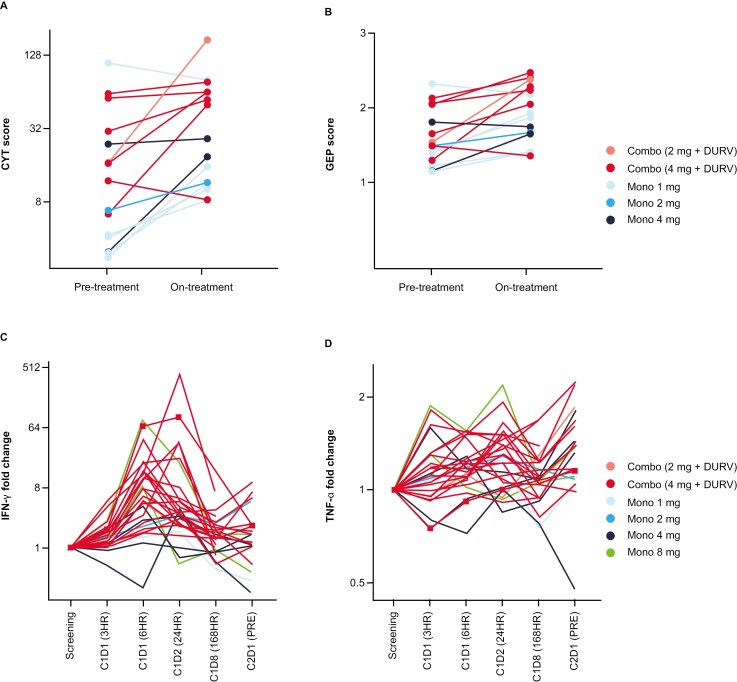
Pharmacodynamic effects of mRNA-2416 in combination with durvalumab. (A) Cytolytic activity (CYT) scores and (B) gene expression profile (GEP) of T-cell-inflamed signature in pretreatment and on-treatment biopsies per patient in arms A and B (*n* = 14). Fold change relative to a baseline of (C) IFNg and (D) TNFa plasma cytokines as measured by electrochemiluminescence in patients treated with mRNA-2416 in both arms A and B, at the indicated doses and timepoints assessed. Cytokine kinetics for the patient with ovarian cancer who had a PR are shown with a square. C, cycle; D, day; DURV, durvalumab; PR, partial response.

Patients in arm B (combination therapy) also demonstrated an upregulation of antitumor immune genes, as suggested by increased T-cell-inflamed signature and cytolytic activity scores post-treatment (Figure 5A and B), and a systemic proinflammatory cytokine response, including elevated levels of interferon γ (IFNγ) and tumor necrosis factor α (TNFα; Figure 5C and D). Peak IFNγ was observed at 24 hours and was elevated in 24/26 patients assessed 24 hours post-treatment. A patient with PR in arm B displayed one of the highest increases in systemic IFNγ after treatment (751 pg/mL at 24 hours; Figure 5C). Although limited by a small sample size, increases in PD-L1 in the TME were observed at a higher magnitude in patients with SD relative to patients with progressive disease, and baseline levels ranged from 0% to 20% positivity in tumor cells in the 4 patients with clinical benefit compared with no expression in most patients with progressive disease (Supplementary Figure S5).

### Pharmacokinetic analyses

Pharmacokinetic analyses of mRNA in serum showed the median (range) t_max_ for mRNA-2416 was 14 hours (3-26 hours) at the 2.0- and 4.0-mg dose levels. Average total serum exposures (AUC_0−t_) were 413,000 and 1,770,000 h*pg/mL following 2.0- and 4.0-mg doses of mRNA-2416, respectively, indicating that both peak and total systemic exposure of mRNA-2416 were sub-proportional to mRNA-2416 dose levels over the 1.0- to 8.0-mg dose range. Average peak serum exposure (C_max_) was 7990 and 20 500 pg/mL following a 2.0- and 4.0-mg dose of mRNA-2416, respectively. Total and peak serum mRNA-2416 exposures were higher when 4.0 mg mRNA-2416 was administered in combination with durvalumab compared with mRNA-2416 alone (Supplementary Table S8).

## Discussion

Results of this phase I/II study provide the first clinical data on the safety, tolerability, and antitumor activity of mRNA-2416, a first-in-class lipid nanoparticle-encapsulated mRNA encoding human OX40L for intratumoral injection, alone and in combination with durvalumab in patients with relapsed/refractory solid tumor malignancies and ovarian cancer. mRNA-2416 was tolerable at all doses assessed—the MTD was not reached—and was associated with increased OX40L protein expression in injected tumors. Although the addition of durvalumab increased the percentage of patients experiencing a TEAE, the majority of events were mild to moderate and no DLTs were reported. The observed safety profile was similar to those previously reported for therapeutics targeting alternative OX40 pathways such as intravenous GSK3174998,^16^ BMS-986178,^17^ MEDI0562,^18,19^ ivuxolimab,^20^ and MOXR0916.^21^ Additionally, there was no added toxicity of durvalumab when given in combination with mRNA-2416. The RDE was 4 mg based on safety and emerging tumor treatment effect at this dose level, with the decision to move forward with this RDE for the fallopian/ovarian expansion cohort based on the low likelihood of metastatic lesions for this histology exceeding the 5-cm cutoff required to accommodate larger volumes of injection.

We provide proof of concept with this first-in-class intratumoral mRNA therapeutic that high local induction of OX40L expression with mRNA-2416 can augment the abundance of T cells and downstream immune modulation within the TME. Patients treated with combination therapy additionally demonstrated more extensive systemic cytokine response, including elevated levels of IFNγ and TNFα, compared with mRNA-2416 monotherapy. Markers of antitumor immunity were upregulated in the TME in both monotherapy and combination therapy groups, with the most extensive modulation of the local immune effector response seen in the combination group, based on cytolytic activity and T-cell-inflamed GEP signatures. The pharmacodynamic data in this study additionally highlight the dual role of the IFNγ-PD-L1 axis in tumor immunity. While IFNγ is known to elicit potent antitumor immunity through T cell activation and Th1 polarization, IFNγ induction of PD-L1 expression may alternatively serve to attenuate local tumor immunity,^22^ providing the rationale for the combinatorial approach with ICI to enhance antitumor immunity.

Despite preclinical data suggesting that immunotherapy should be effective in epithelial ovarian cancer,^23^ clinical successes with immune-based therapies have been modest.^24,25^ Studies have shown the importance of the TME in the facilitation of immune tolerance, with induction of proinflammatory cytokines and activation of immune effector cells needed to overcome immunosuppressive mechanisms promoting refractory disease.^26,27^ Based on observations of preliminary activity in patients with ovarian cancer, evidence of modulation of the TME, and upregulation of PD-L1 expression in tumor biopsies after injection of mRNA-2416 alone, we pursued evaluation of the combination of mRNA-2416 and durvalumab in an expansion cohort in patients with advanced metastatic fallopian/ovarian cancer to explore preliminary activity in this setting. While RECIST v1.1-defined responses were not seen in this cohort and primary efficacy endpoints were not met, we detected evidence of tumor shrinkage in both injected and non-injected tumors (Figure 3). The observation of tumor shrinkage in both the injected tumor and surrounding non-injected lesion was most notable in a patient with durable SD supports the notion that local induction of OX40L, followed by expansion of tumor-specific T cells, may broaden the immune response beyond the direct field of injection.

Limitations of this study include those inherent to an intratumoral platform where the volume of injection placed constraints on the size of the lesion amenable to injection. In this study, the MTD was not reached and the RDE was determined based on the expected low likelihood of large (>5 cm) metastatic lesions in ovarian cancers. More importantly, the patient population consisting of chemotherapy-refractory disease may have impacted clinical activity. In this study, patients received a median of 4 prior lines of therapy (range: 1-18) and more than half of patients had also received prior immune ICIs (59% in arm A; 45% in arm B). Additionally, the observed modest responses, despite pharmacodynamic evidence of enhanced OX40L expression, likely reflect the multiple immune resistance mechanisms in the advanced refractory setting. Finally, emerging studies suggest that simultaneous PD-1 axis blockade added at the initiation of OX40 therapy may negate the effects of OX40 agonism through induction of T-cell apoptosis while PD-1 blockade following OX40 agonism did not alter the antitumor effects.^28^ In these studies, tumor-infiltrating antigen-specific CD8 + T cells were diminished due to induction of T-cell apoptosis in both the tumor and the periphery with concurrent administration, suggesting serial administration may be superior to concurrent administration of the combination and has implications for the design of future combinatorial strategies.

## Conclusions

In summary, this first-in-human study of mRNA-2416, alone and in combination with durvalumab, shows that mRNA-2416 was tolerable across all dose levels, and the MTD was not reached. Although predefined primary efficacy endpoints were not met, pharmacodynamic analyses provide evidence of the mechanism of action and support continued research into mRNA-based therapeutics to address the unmet challenge of difficult-to-treat malignancies. Additional research is needed to further elucidate biomarkers of response and identification of the subset of patients most likely to benefit. Future studies should also evaluate alternative administration schedules, including serial versus concurrent administration of ICIs. The addition of proinflammatory cytokines to OX40 agonism may additionally serve to further ignite and transform the immune suppressive TME into a more productive immune response, particularly in the immune checkpoint-refractory setting. This approach is currently being evaluated in a phase I study of mRNA-2752 (OX40L/IL-23/IL-36γ) and is expected to be informative for future development of therapeutic options in the immune checkpoint refractory setting.^29^

## Supplementary Material

oyaf115_suppl_Supplementary_Figures_S1-S5_Tables_S1-S8

## Data Availability

Access to patient-level data presented in this article and supporting clinical documents with external researchers who provide methodologically sound scientific proposals will be available upon reasonable request for products or indications that have been approved by regulators in the relevant markets and subject to review from 24 months after study completion. Such requests can be made to 325 Binney Street, Cambridge, MA, 02142 USA or data_sharing@modernatx.com. A materials transfer and/or data access agreement with the sponsor will be required for accessing shared data. All other relevant data are presented in the paper. The protocol is available online at ClinicalTrials.gov: https://clinicaltrials.gov/study/NCT03323398.
