# Supplementary material for: First-in-human phase I/II, open-label study of mRNA-2416 alone or combined with durvalumab in patients with advanced solid tumors and ovarian cancer
Source: Oncologist. 2025 Jun 14;30(6):oyaf115. doi: 10.1093/oncolo/oyaf115 (PMC12166121; doi:10.1093/oncolo/oyaf115)
Supplement: oyaf115_suppl_Supplementary_Figures_S1-S5_Tables_S1-S8 [file oyaf115_suppl_supplementary_figures_s1-s5_tables_s1-s8.pdf]

Supplementary Data

Supplementary Text

Dose escalation

For both arms, dose escalation was conducted using a standard 3+3 study design to determine safety and tolerability of the study therapy.

Dose confirmation

For both arms, once the expected maximum tolerated dose (MTD)/recommended dose for expansion (RDE) was cleared in the dose escalation part, dose confirmation of the MTD/RDE was conducted in ≥3 patients with visceral lesions. The purpose of dose confirmation was to confirm that the dose level determined for patients with accessible lesions is also appropriate for patients with visceral lesions. Dose confirmation was conducted similarly to the dose escalation part as described above.

Dose expansion

Once the MTD and/or RDE was determined in the dose escalation/confirmation parts, patients were enrolled in the dose expansion part in order to assess the preliminary antitumor activity of mRNA-2416 in combination with durvalumab in patients with fallopian/ovarian cancer. The dose expansion part sought to enroll 41 evaluable patients using a Simon two-stage design, with enrollment of 15 patients during the first stage

and 26 patients during the second stage if 2 or more responses were seen during the first stage.

Study stopping rules during dose expansion

The dose-limiting toxicity (DLT) rate was computed during the dose expansion part to implement study stopping rules. The DLT rate was calculated as events occurred and was defined as the ratio of the total number of patients with a DLT at the MTD/RDE divided by the total number of patients enrolled at that dose level who were evaluable for DLTs. If the DLT rate was ≥33%, a temporary study suspension of enrollment at the MTD/RDE was required to review available safety information and determine whether additional patients should be enrolled at that dose level. The Safety Review Committee determined whether further enrollment at that specific dose level was permitted or if a lower dose level should commence.

Sample size considerations

The null hypothesis that the true ORR was 0.05 was tested, and with the sample size of 13 and 14 for phases I and II, respectively, this design provided an overall one-sided alpha value of 0.05 and statistical power of 0.80 when the true ORR was 0.20. For arm B, the null hypothesis that the true ORR was 0.1 was tested, and the sample size of 15 and 26 for phases I and II, respectively, provided an overall one-sided alpha value of 0.05 and statistical power of 0.80 when the true ORR was 0.25.

Supplementary Table S1 Definitions of MTD and DLT

Maximum tolerated dose (MTD)	The MTD was defined as the highest dose of mRNA-2416 alone and in combination with durvalumab at which a DLT was seen in <2 of 6 (<33%) patients during the first 28 days of active treatment.
Dose-limiting toxicity (DLT)	DLT is defined as an adverse event (AE) or abnormal laboratory value assessed as unrelated to disease, disease progression, intercurrent illness, or concomitant medications; that is at least possibly related to the study therapy; and occurring within the first 28 days of study therapy.<br>For patients in arm A receiving mRNA-2416, the following toxicities were considered a DLT:<br>• All grade 3 AEs with the exception of the following:<br>o Grade 3 thrombocytopenia lasting <7 days<br>o Grade 3 neutropenia without fever or lasting <7 days<br>• Any grade 4 or 5 toxicity<br>• Death due to disease progression is not considered a DLT<br>For patients in arm B receiving mRNA-2416 in combination with durvalumab, the following toxicities were considered a DLT:<br>• Any grade ≥3 immune or non-immune AE that is at least possibly related to the investigational product or investigational regimen with two exceptions:<br>o Any grade of vitiligo or alopecia<br>Hematologic toxicity:<br>• Grade ≥3 neutropenia complicated by fever >38.3°C<br>• Grade 4 neutropenia >7 days<br>• Grade ≥3 thrombocytopenia with significant bleeding<br>• Grade 4 thrombocytopenia (regardless of duration)<br>• Grade 4 anemia (regardless of duration)

Supplementary Table S2 Patient characteristics for the phase II study

Characteristic	Phase II study
	**Arm B (n=16)**
Age, years, median (range)	63 (54–78)
Female sex, n (%)	16 (100)
Race, n (%)	
Black	—
Other	2 (13)
White	14 (88)
Ethnicity, n (%)	
Hispanic or Latino	—
Not Hispanic or Latino	16 (100)
Time since initial diagnosis to first dose, months, median (range)*	71.7 (13.8–179.1)
Cancer types, n (%)	
Fallopian/ovarian	16 (100)
ECOG performance status, n (%)	
0	5 (31)
1	11 (69)
2	—

*Time from initial diagnosis to first dose in months is calculated as (date of first dose of study drug − date of initial diagnosis + 1)/30.4375.

ECOG, Eastern Cooperative Oncology Group.

Supplementary Table S3 Safety summary of treatment emergent adverse events

Characteristic	Arm A (n=39)	Arm B (n=22)	Overall population (N=61)
Any TEAE	38 (97)	22 (100)	60 (98)
TEAE with NCI CTCAE ≥ grade 3	24 (62)	19 (86)	43 (70)
mRNA-2416-related TEAE	31 (80)	17 (77)	48 (79)
Durvalumab-related TEAE	—	15 (68)	15 (25)
Serious TEAE	19 (49)	16 (73)	35 (57)
TEAE leading to discontinuation of mRNA-2416	5 (13)	3 (14)	8 (13)
TEAE leading to discontinuation of durvalumab	—	4 (18)	4 (7)
TEAE of special interest for durvalumab	10 (26)	13 (59)	23 (38)
TEAE leading to death	7 (18)	4 (18)	11 (18)

Data are n (%).

A TEAE is defined as any AE that has newly appeared, increased in frequency, or worsened in severity occurring on or after the first dose of the study drug.

AE, adverse event; NCI CTCAE, National Cancer Institute Common Terminology Criteria for Adverse Events; TEAE, treatment-emergent adverse event.

Supplementary Table S4 Treatment-related TEAEs by preferred term occurring with frequency ≥5% for arm A or B

	**Arm A**							**Arm B**					
	**Dose escalation**					**Dose confir-mation**	**Total (N=39)**	**Dose escala-tion**	**Dose confir-mation**	**Dose expansion**			**Total (N=22)**
	**1 mg (n=11)**	**2 mg (n=12)**	**4 mg (n=12)**	**8 mg (n=3)**	**Sub-total (n=38)**	**8 mg (n=1)**		**4 mg (n=3)**	**4 mg (n=3)**	**2 mg (n=1)**	**4 mg (n=15)**	**Sub-total (n=16)**	
**Patients with ≥1 durvalumab-related TEAE**	—	—	—	—	—	—	—	1 (33.3)	2 (66.7)	1 (100)	11 (73.3)	12 (75.0)	15 (68.2)
Fatigue	—	—	—	—	—	—	—	0	1 (33.3)	0	5 (33.3)	5 (31.3)	6 (27.3)
Pyrexia	—	—	—	—	—	—	—	0	0	0	5 (33.3)	5 (31.3)	5 (22.7)
Nausea	—	—	—	—	—	—	—	1 (33.3)	1 (33.3)	0	3 (20.0)	3 (18.8)	5 (22.7)
Vomiting	—	—	—	—	—	—	—	0	0	0	2 (13.3)	2 (12.5)	2 (9.1)
Arthralgia	—	—	—	—	—	—	—	0	0	0	2 (13.3)	2 (12.5)	2 (9.1)
Arthritis	—	—	—	—	—	—	—	0	0	0	2 (13.3)	2 (12.5)	2 (9.1)
Myalgia	—	—	—	—	—	—	—	0	0	0	2 (13.3)	2 (12.5)	2 (9.1)
Increased aspartate aminotransferase	—	—	—	—	—	—	—	0	0	0	2 (13.3)	2 (12.5)	2 (9.1)

Data are n (%). A TEAE is defined as any adverse event that has newly appeared, increased in frequency, or worsened in severity occurring on or after the first dose of the study drug. Patients may have more than one event per system organ class and preferred term. At each level of patient summarization, a patient is counted once if the patient reported one or more events. Adverse events were coded with Medical Dictionary for Regulatory Activities Version 24.1.

TEAE, treatment-emergent adverse event.

Supplementary Table S5 Safety summary by dose level for arm A

	Dose escalation					Dose confirmation	Total (N=39)
	**1 mg (n=11)**	**2 mg (n=12)**	**4 mg (n=12)**	**8 mg (n=3)**	**Sub-total (n=38)**	**8 mg (n=1)**	
TEAE	10 (91)	12 (100)	12 (100)	3 (100)	37 (97.4)	1 (100)	38 (97.4)
TEAE with NCI CTCAE ≥grade 3	5 (45.5)	8 (66.7)	9 (75.0)	1 (33.3)	23 (60.5)	1 (100)	24 (61.5)
mRNA-2416–related TEAE	6 (54.5)	11 (91.7)	11 (91.7)	3 (100)	31 (81.6)	0 (0)	31 (79.5)
Serious TEAE	3 (27.3)	6 (50.0)	7 (58.3)	2 (66.7)	18 (47.4)	1 (100)	19 (48.7)
TEAE leading to discontinuation of mRNA-2416	2 (18.2)	2 (16.7)	1 (8.3)	0	5 (13.2)	0	5 (12.8)
TEAE leading to death	1 (9.1)	1 (8.3)	3 (25.0)	1 (33.3)	6 (5.8)	1 (100)	7 (17.9)

Data are n (%).

A treatment-emergent adverse event is defined as any adverse event that has newly appeared, increased in frequency, or worsened in severity occurring on or after the first dose of the study drug.

AE, adverse event; NCI CTCAE, National Cancer Institute Common Terminology Criteria for Adverse Events; TEAE, treatment-emergent adverse event.

Supplementary Table S6 Safety summary by dose level for arm B

	Dose escalation	Dose confirmation	Dose expansion			Total (N=22)
	4 mg (n=3)	4 mg (n=3)	2 mg (n=1)	4 mg (n=15)	Sub-total (n=16)	
TEAE	3 (100)	3 (100)	1 (100)	15 (100)	16 (100)	22 (100)
TEAE with NCI CTCAE ≥grade 3	2 (66.7)	3 (100)	1 (100)	13 (86.7)	14 (87.5)	19 (86.4)
mRNA-2416–related TEAE	1 (33.3)	3 (100)	1 (100)	12 (80.0)	13 (81.3)	17 (77.3)
Durvalumab-related TEAE	1 (33.0)	2 (66.7)	1 (100)	11 (73.3)	12 (75.0)	15 (68.2)
Serious TEAE	2 (66.7)	2 (66.7)	1 (100)	11 (73.3)	12 (75.0)	16 (72.7)
TEAE leading to discontinuation of mRNA-2416	1 (33.0)	0 (0)	1 (100)	1 (6.7)	2 (12.5)	3 (13.6)
TEAE leading to discontinuation of durvalumab	1 (33.3)	0 (0)	1 (100)	2 (13.3)	3 (18.8)	4 (18.2)
TEAE of special interest for durvalumab	0 (0)	3 (100)	1 (100)	9 (60.0)	10 (62.5)	13 (59.1)
TEAE leading to death	0 (0)	0 (0)	0 (0)	4 (26.7)	4 (25.0)	4 (18.2)

Data are n (%).

A TEAE is defined as any adverse event that has newly appeared, increased in frequency, or worsened in severity occurring on or after the first dose of the study drug.

AE, adverse event; NCI CTCAE, National Cancer Institute Common Terminology Criteria for Adverse Events; TEAE, treatment-emergent adverse event.

Supplementary Table S7 Objective response rate, disease control rate, and progression-free survival in both treatment arms

Endpoint	RECIST v1.1		irRC and LYRIC	
	**Arm A**	**Arm B**	**Arm A**	**Arm B**
Objective response rate, % (95% CI)*	0.0 (0.0–11.9)	5.3 (0.1–26.0)	0.0 (0.0–11.9)	5.3 (0.1–26.0)
Disease control rate, % (95% CI)*	31.0 (15.3–50.8)	15.8 (3.4–39.6)	—	—
Progression-free survival, days, median (95% CI); [range]	60.0 (50.8–108.0); [29–179]	50.0 (38.0–55.0); [11–117]	60.0 (50.0–179.0); [29–239]	52.0 (38.0–75.0); [11–120]

*95% CI based on Clopper–Pearson exact test.

CI, confidence interval; irRC, immune-related response criteria; LYRIC, Lymphoma Response to Immunomodulatory Therapy Criteria; RECIST, Response Evaluation Criteria in Solid Tumors.

Supplementary Table S8 Comparison of peak and total serum exposure between mRNA-2416 with and without durvalumab

	**Period 1: mRNA-2416**		**Period 3: mRNA-2416 with durvalumab**	
**2-mg dose**	**Part A (n=2)**	**Part B (n=3)**	**Part A (n=NC)**	**Part B (n=NC)**
C_{max}, pg/mL	7,990 ± 5,250 (66)	NC	NC	NC
t_{max}, hours	14.0 (5.85, 22.13)	NC	NC	NC
$AUC_{0–t}$, pg/mL*hour	413,000 ± 45,800 (11)	NC	NC	NC
**4-mg dose**	**Part A (n=8)**	**Part B (n=3)**	**Part A (n=NC)**	**Part B (n=3)**
C_{max}, pg/mL	20,500 ± 24,100 (118)	24,700 ± 31,300 (127)	NC	83,300 ± 108,000 (129)
t_{max}, hours	14.4 (3.00, 214.63)	23.5 (2.95, 26.00)	NC	3.23 (2.98, 5.60)
$AUC_{0–t}$, pg/mL*hour	1,770,000 ± 218,000 (123)	1,550,000 ± 1,890,000 (122)	NC	5,140,000 ± 6,360,000 (124)

t_{max} is reported as median (min, max) while C_{max} and AUC are reported as mean ± STD (%CV).

AUC, area under the curve; $AUC_{0–t}$, AUC over a dosing interval; C_{max}, maximum observed concentration; CV, coefficient of variation; NC, not calculated; STD, standard deviation; t_{max}, time to peak concentration.

Supplementary Figure S1.

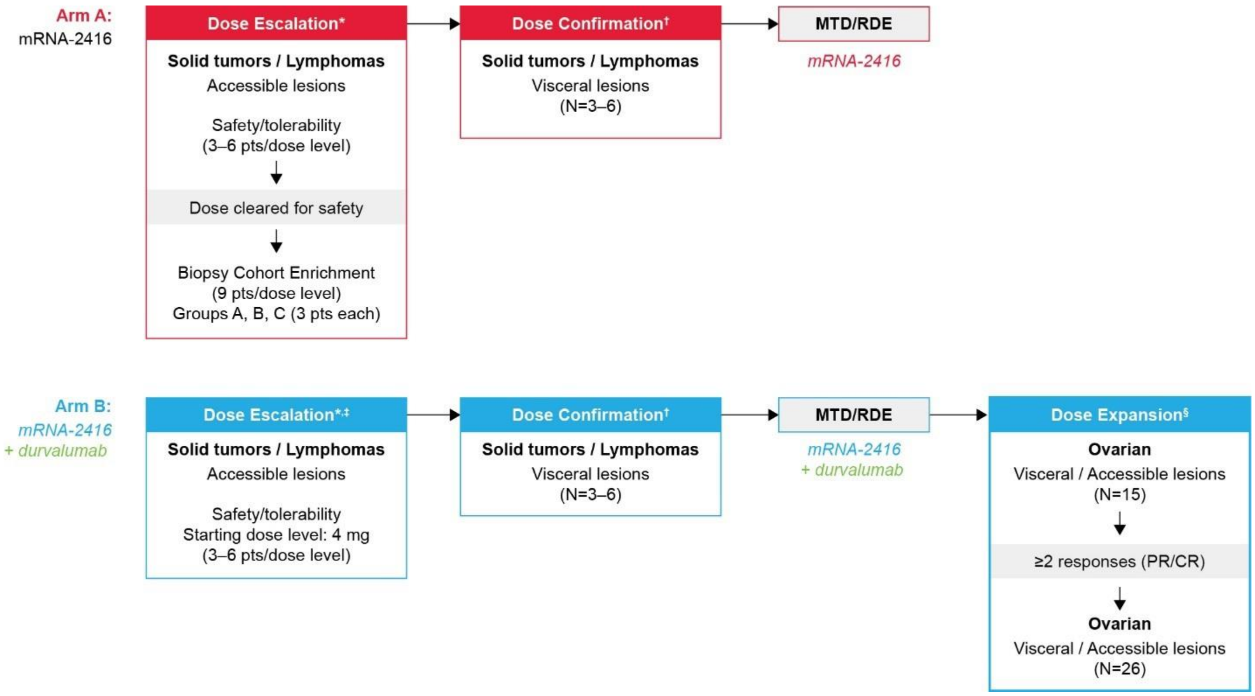

Study design for the phase I (dose escalation and confirmation) and phase II (dose expansion) studies.

*Dose escalation: 3+3 model (DLT window was 1 cycle (28 days).

†Dose confirmation: Confirmation of MTD/RDE in visceral lesions after clearance of MTD/RDE in accessible lesions.

‡Dose escalation in arm B: Began once expected MTD/RDE cleared in arm A dose escalation.

§Dose expansion: Simon two-stage model.

DLT, dose-limiting toxicity; MTD, maximum tolerated dose; RDE, recommended dose for expansion.

Supplementary Figure S2.

A

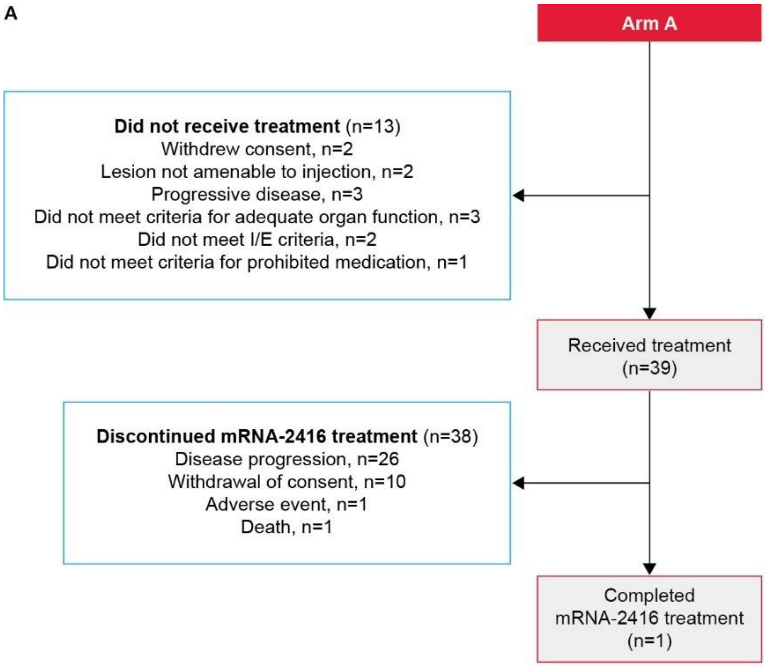

B

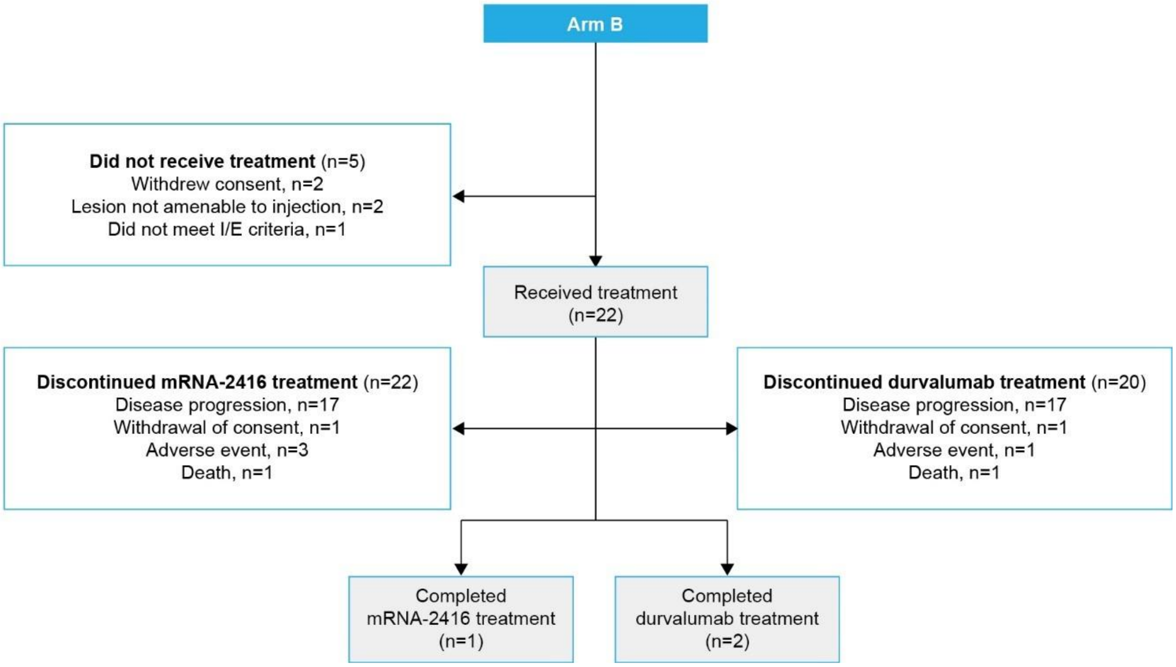

Flow chart of patients randomized to arm A or B. I/E, inclusion/exclusion.

Supplementary Figure S3.

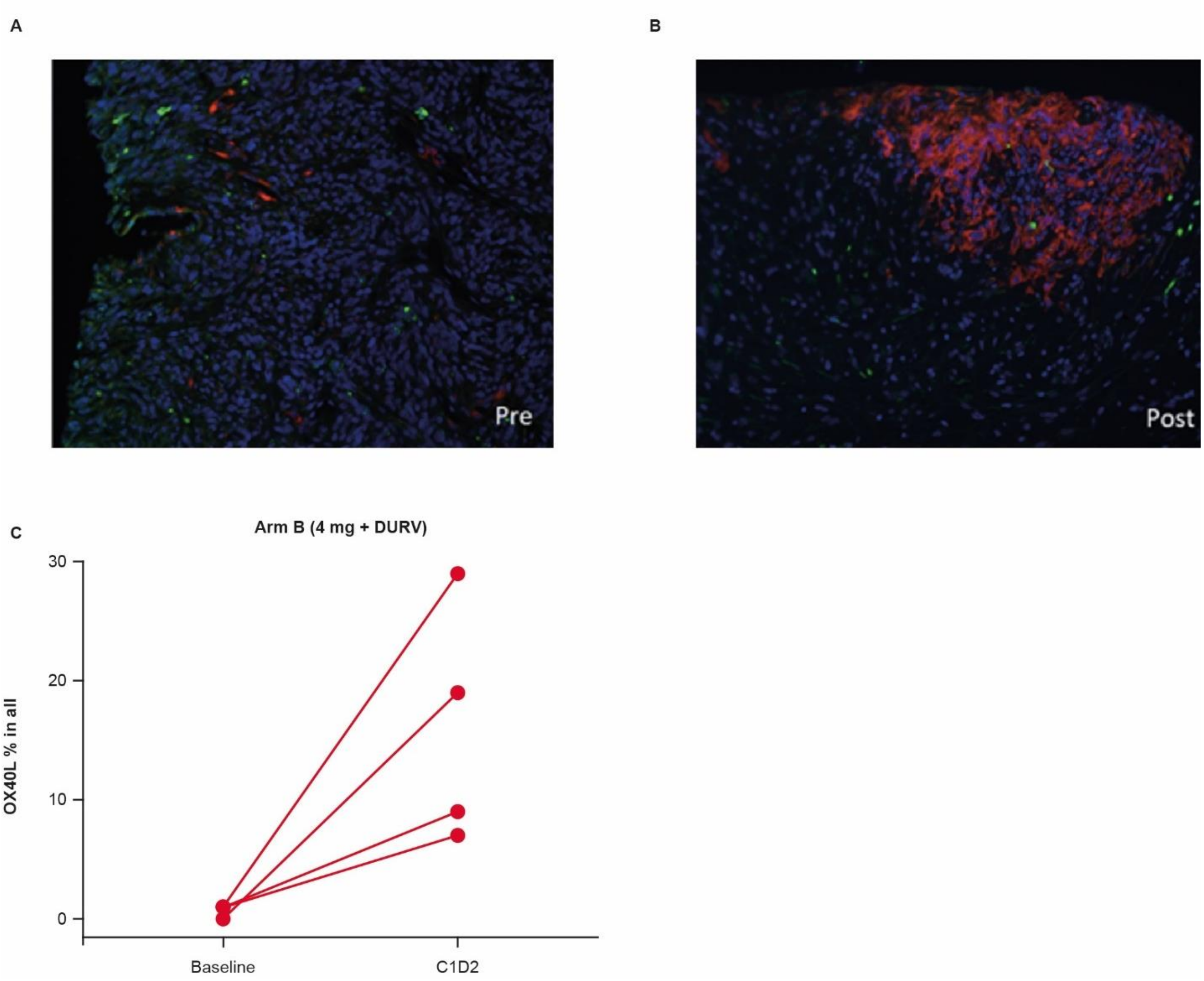

OX40L protein expression by multiplex quantitative immunofluorescence. (A) Pre– and (B) post–mRNA-2416 injection representative OX40L expression (OX40L, red; cytokeratin (CK), green; DAPI, blue). (C) OX40L protein expression 24–48 hours post–mRNA-2416 treatment in arm B, with 4.0-mg mRNA-2416 dose, paired pre-treatment versus on-treatment biopsies. "% in all" indicates quantification in both tumor and peri-tumor stromal regions (CK+ & CK−). CK− reflect stromal cells, including immune cells; CK+ cells reflect tumor cells.

DURV, durvalumab; C1D2, cycle 1, day 2.

Supplementary Figure S4.

Un-injected lesions at C1D22–28 (7–13 days post-treatment) < **Injected** lesions at C1D2 or C2D2 (1-day post-treatment / during acute phase response)

		Group A					Group B & C								
		(1A, ESN)	(1A, NSCLC)	(2A, OVCA)	(3A, CCA)	(3A, OVCA)	(1B, HNSCC)	(1B, OVCA)	(1B, PRAD)	(1C, HNSCC)	(1C, SARC)	(2B, HNSCC)	(3B, BRCA)	(3B, PRCA)	(3C, OVCA)
OX40L/OX40	TNFSF4	3446.65	1.27	0.88	1.80	23.58	2.57	806.78	6834.04	15.80	2782.14	3.08	2.77	442.55	0.86
	TNFRSF4	0.57	1.25	0.74	0.87	0.75	1.06	1.90	1.18	1.69	0.68	2.70	0.84	0.80	0.85
PD-L1/PD-1	CD274	1.31	0.79	0.43	0.56	1.19	0.98	4.47	1.21	2.38	16.69	1.07	3.08	2.53	1.26
	PDCD1LG2	1.65	1.05	0.64	0.71	2.26	0.67	1.73	2.86	2.88	1.18	1.53	2.33	1.03	0.81
	PDCD1	0.58	1.18	0.59	0.82	1.21	0.83	1.25	1.17	1.52	3.02	2.56	1.97	0.78	0.79
T-cell abundance	CD4	0.65	1.20	0.92	0.89	1.49	0.72	3.55	3.12	2.18	0.90	1.44	1.39	0.51	0.82
	CD8A	0.51	0.99	0.56	1.33	1.04	0.74	5.17	1.18	4.55	3.67	1.96	6.29	0.57	0.91
T-cell inflammation	GZMB	0.46	0.87	0.42	0.54	2.93	0.57	9.80	1.51	3.15	2.88	1.19	1.54	0.67	1.94
	TNF	0.77	1.04	1.03	1.10	0.84	1.41	2.92	1.46	2.28	2.35	1.03	1.09	2.07	1.19
	IFN-γ	0.58	1.04	0.79	0.81	1.17	0.89	2.62	0.74	2.50	5.15	1.16	1.53	0.66	0.90
	CD69	1.06	1.14	1.81	1.23	2.16	1.24	5.34	2.16	11.79	19.10	1.92	4.33	2.75	0.68
	ICOS	0.87	1.00	0.89	0.97	1.01	0.82	3.16	1.21	2.18	3.03	1.32	2.78	0.93	0.60
DC abundance	BATF3	0.84	0.95	0.66	0.87	0.81	1.86	1.54	4.15	1.78	2.96	1.12	1.99	1.60	1.03
	CLEC9A	1.33	1.19	0.72	0.95	0.93	0.54	1.12	1.20	1.08	1.09	1.46	1.77	0.62	0.70
	XCR1	1.11	0.82	0.72	0.78	0.94	0.52	2.18	4.04	0.25	1.24	2.08	2.07	0.73	0.64
	CCL3	0.65	1.16	0.91	0.83	0.46	0.55	8.43	2.12	5.28	3.20	3.17	0.74	3.57	1.30
	CCL4	0.56	0.92	1.25	0.82	1.05	0.79	5.78	1.52	5.06	5.60	1.49	1.64	1.59	1.10
	CXCL9	1.69	1.33	0.06	0.30	13.80	0.54	7.90	0.38	5.17	41.38	0.51	0.49	0.42	0.63
	CXCL10	1.70	1.55	0.13	0.37	2.78	1.20	3.36	0.63	3.12	751.95	1.14	2.33	2.30	1.38
	CD80	0.99	1.10	0.65	0.93	0.69	0.95	3.11	3.96	1.90	6.42	1.66	0.93	2.06	0.82
	CD86	1.04	1.23	0.63	1.01	1.98	1.01	3.72	4.18	2.23	3.53	1.47	1.23	1.30	0.65
	FLT3LG	1.30	0.82	0.63	1.49	0.78	0.82	1.51	2.99	1.36	1.32	1.02	1.82	0.66	1.30
	LAMP3	1.31	0.93	0.40	0.52	3.67	1.73	0.76	0.90	3.54	4.80	1.70	2.65	3.76	1.22
	FLT3	1.14	1.09	0.66	0.64	1.71	0.63	3.64	1.01	1.01	1.64	1.33	4.68	0.76	0.81

Transcriptional changes post–mRNA-2416 treatment in injected and non-injected tumors. Marker genes of T-cell and DC abundance and activation[1-3] were collected from the literature. Fold change of gene expression (TPM, with 1 offset) was calculated from pre- and post-treatment samples for each gene. Individual value fields are heatmapped, with red to blue indicating elevated to reduced expression, respectively, per case post-treatment. TNFSF4=OX40L; TNFRSF4=OX40; CD274=PD-L1; PDCD1LG2=PD-L2; PDCD1=PD-1.

BRCA, breast cancer; C, cycle; CCA, cholangiocarcinoma; D, day; DC, dendritic cell; ENB, esthesioneuroblastoma; HNSCC, head and neck squamous cell carcinoma; NSCLC, non-small cell lung cancer; OVCA, ovarian cancer; PRAD, prostate adenocarcinoma; PRCA, prostate cancer; SARC, sarcoma; TPM, transcripts per kilobase million.

Supplementary Figure S5.

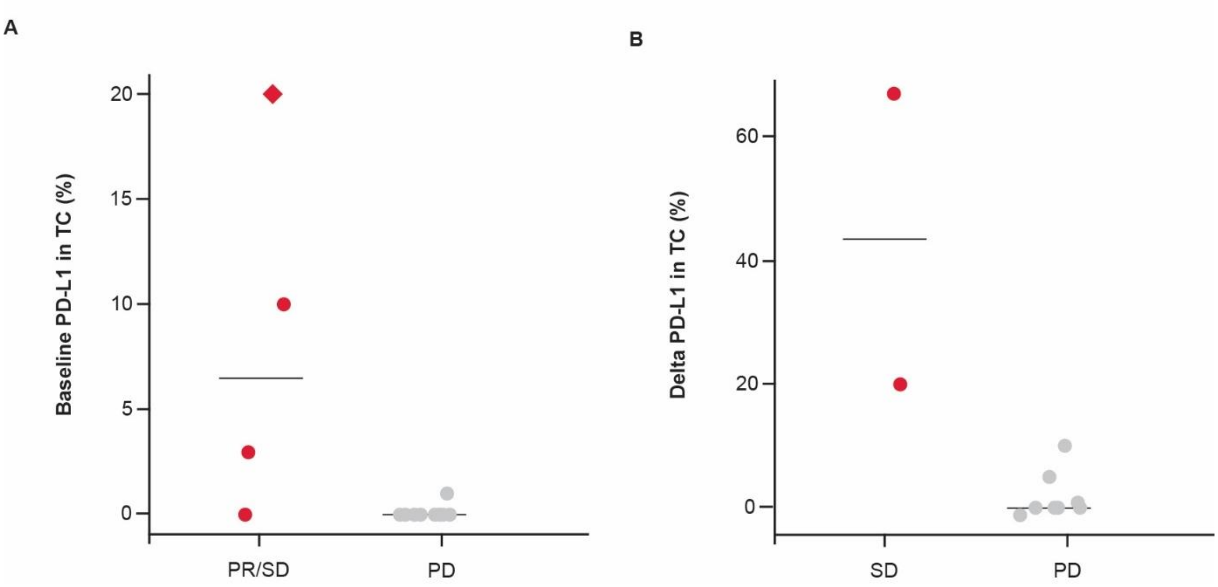

Protein-level PD-L1 expression in TCs by SP263 IHC shown in (A) as the frequency at baseline, and (B) as the change on treatment relative to baseline (delta = % on treatment minus % pre-). PD-L1 expression shown for the patient with ovarian cancer who had a PR is shown by a diamond. IHC, immunohistochemistry; PR, partial response; SD, stable disease; PD, progressive disease; TC, tumor cells.

References

1. Danaher P, Warren S, Dennis L, D'Amico L, White A, Disis ML, *et al*. Gene expression markers of Tumor Infiltrating Leukocytes. J Immunother Cancer **2017**;5:18 doi 10.1186/s40425-017-0215-8.

2. Simoni Y, Becht E, Fehlings M, Loh CY, Koo SL, Teng KWW, *et al*. Bystander CD8(+) T cells are abundant and phenotypically distinct in human tumour infiltrates. Nature **2018**;557(7706):575-9 doi 10.1038/s41586-018-0130-2.

3. Hewitt SL, Bai A, Bailey D, Ichikawa K, Zielinski J, Karp R, *et al*. Durable anticancer immunity from intratumoral administration of IL-23, IL-36gamma, and OX40L mRNAs. Sci Transl Med **2019**;11(477):eaat9143 doi 10.1126/scitranslmed.aat9143.
